# Being born in autumn or winter is associated with asthma and allergic rhinitis in Finland

**DOI:** 10.1002/clt2.12383

**Published:** 2024-07-19

**Authors:** Riikka Hänninen, Aada Murtomäki, Fanni Svärd, Aarno Dietz, Paulus Torkki, Jari Haukka, Mikko Nuutinen, Sanna Toppila‐Salmi

**Affiliations:** ^1^ Department of Otorhinolaryngology University of Eastern Finland Joensuu Kuopio Finland; ^2^ Deparment of Allergology Inflammation Center Skin and Allergy Hospital Helsinki University Hospital and University of Helsinki Hospital District of Helsinki and Uusimaa Helsinki Finland; ^3^ Department of Otorhinolaryngology Kuopio University Hospital Wellbeing Services County of North Savo Kuopio Finland; ^4^ Department of Public Health University of Helsinki Helsinki Finland; ^5^ Haartman Institute University of Helsinki Helsinki Finland

**Keywords:** AERD, allergic rhinitis, allergy, asthma, chronic rhinosinusitis, N‐ERD, non‐steroidal anti‐inflammatory drug exacerbated respiratory disease, rhinitis, season of birth

## Abstract

**Background:**

Our population‐based study has previously shown that being born in winter or spring was associated with adult‐onset asthma. The aim was to study if season of birth (SOB) is associated with airway allergy and related diseases: NSAID exacerbated respiratory disease (N‐ERD), asthma, allergic rhinitis (AR), nonallergic rhinitis (NAR), chronic rhinosinusitis with nasal polyps (CRSwNP) and CRS without nasal polyps (CRSsNP) in Finland.

**Methods:**

A randomly sampled retrospective registry‐based follow‐up data (*n* = 74,868) of patients visiting Hospital District of Helsinki and Uusimaa (HUS) in Finland was used. The birth date, sex, visit date and comorbidities were collected from electronic health record data during visits from 2005 to 2019.

**Results:**

The mean (SD, range) age of the sample was 34.53 (25.47, 0–102) years, with 48.7 % being men. We divided the whole population in four groups based on the season they were born (SOB‐groups). When observing these four SOB‐groups, the proportion of those having asthma was 43.1%, 42.1%, 41.1%, 42.7%, in winter, spring, summer, and autumn SOB‐groups, respectively. The proportion of those having AR was 12.6%, 12.0%, 10.7%, 12.1%, respectively. When having summer as a reference, being born in any other time of year was significantly associated with AR and, being born in autumn or winter was associated with asthma. No significant association was observed in CRS or N‐ERD or NAR groups in adjusted models.

**Conclusions:**

The study suggests that early life immunological events may have a role a role in pathogenesis of asthma and AR. As no association was observed between SOB and CRSsNP, CRSwNP, N‐ERD or NAR, further studies on this are warranted.

To the Editor,

In populations exposed to strong variation of luminosity, season of birth (SOB) has been shown to be associated with several non‐communicable diseases in children and adults.^1^, ^2^ Our previous population‐based study has demonstrated an association between SOB and the adult‐onset asthma in Finland.^3^ The aim was to study if SOB is associated with airway allergy and related diseases: NSAID exacerbated respiratory disease (N‐ERD), asthma, allergic rhinitis (AR), nonallergic rhinitis (NAR), chronic rhinosinusitis with nasal polyps (CRSwNP) and CRS without nasal polyps (CRSsNP) in Finland.

A randomly sampled retrospective registry‐based follow‐up data (*n* = 74,868) of patients having otorhinolaryngological and respiratory diagnoses at the Hospitals of Hospital District of Helsinki and Uusimaa (Table S1) were used. The ethics committee of the Hospital District approved the study (no. 31/13/03/00/2015) and decided that there is no need of the written consent. We collected the following covariates by using ICD‐10 codes and keyword searches of electronic health records (EHR) during the visits from 2005 to 2019 as previously described^4^: gender, age, birth date, NAR (J31.), AR (J30.), CRSwNP (J33.), CRSsNP (J32. + no J33. + no existing EHR of nasal polyps), asthma (J45.), allergy (J45.0, or J30., or EHR “allergy”), N‐ERD (keyword search; Table S2). Statistical analyses were conducted by using Python (version 3.10) and statsmodels package (version 0.14.0).^5^ Logistic regression models were adjusted by sex and age, and born season was divided into winter (December‐February), spring (March‐May), summer (June‐August) and autumn (September‐November). Summer was the reference season is all Logistic regression models.

The mean (SD, range) age of the sample was 34.53 (25.47, 0–102) years, with 48.7% being men. Venn diagrams show the absolute number of subjects having overlapping diagnoses of rhinitis/rhinosinusitis and closely related diseases (Figure 1). We divided the whole population in four groups based on the season they were born (SOB‐groups). When observing these four SOB‐groups, the proportion of those having asthma was 43.1%, 42.1%, 41.1%, 42.7%, in winter, spring, summer, and autumn SOB‐groups, respectively (Table S3). The proportion of those having AR was 12.6%, 12.0%, 10.7%, 12.1%, respectively (Table S3). Being born in autumn or winter was statistically significantly associated with AR or with asthma (Table 1). In addition, being born in spring was associated with AR (Table 1).

**FIGURE 1:**
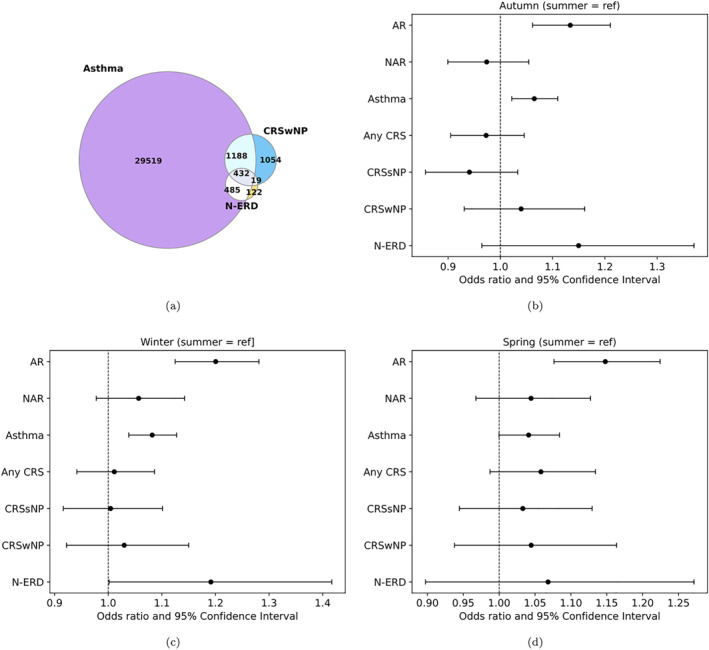
(A) A Venn diagram shows the absolute number of subjects having overlapping diagnoses. AR = allergic rhinitis; CRSsNP = chronic rhinosinusitis without nasal polyps, CRSwNP = chronic rhinosinusitis with nasal polyps; NAR = non‐allergic rhinitis; N‐ERD, non‐steroidal anti‐inflammatory drug‐exacerbated respiratory disease. (B–D) Forest plots of the risk of being born in (B) autumn (C) winter and (D) spring as compared with summer.

**TABLE 1:** The association of season of birth with different diseases.

Disease	Autumn		Winter		Spring	
	OR (95% CI)	*p*	OR (95% CI)	*p*	OR (95% CI)	*p*
AR	1.13 (1.06–1.21)	**<0.000**	1.2 (1.12–1.28)	**<0.000**	1.15 (1.08–1.22)	**<0.000**
NAR	0.97 (0.9–1.05)	0.513	1.06 (0.98–1.14)	0.164	1.04 (0.97–1.13)	0.266
Asthma	1.06 (1.02–1.11)	**0.003**	1.08 (1.04–1.13)	**<0.000**	1.04 (1.0–1.08)	0.051
Any CRS	0.97 (0.9–1.05)	0.453	1.01 (0.94–1.09)	0.756	1.06 (0.99–1.13)	0.111
CRSsNP	0.94 (0.86–1.03)	0.202	1.0 (0.92–1.1)	0.923	1.03 (0.94–1.13)	0.478
CRSwNP	1.04 (0.93–1.16)	0.490	1.03 (0.92–1.15)	0.601	1.04 (0.94–1.16)	0.428
N‐ERD	1.15 (0.96–1.37)	0.120	1.19 (1.0–1.42)	0.048	1.07 (0.9–1.27)	0.459

*Note*: *p* values by binary logistic regression adjusted by sex and age. CI = confidence intervals. Reference value was summer (Jun‐Aug) in all models. The information extraction method from clinical texts was based on two separate methods that have been previously described.^6^ In brief, we first searched directly for ICD‐10 codes from the clinical texts. Secondly, we searched keywords related to basic diseases (such as “diabetes” and “N‐ERD”). CRS = chronic rhinosinusitis; CRSsNP= CRS without nasal polyps (J32. and no J33.); CRSwNP = CRS with nasal polyps; NAR (nonallergic rhinitis J31); AR (allergic rhinitis J30); N‐ERD=NSAID exacerbated respiratory disease. N‐ERD diagnosis was based on a positive medical history of patient‐reported wheezing, coughing or naso‐ocular symptoms after taking non‐steroidal anti‐inflammatory drugs (NSAIDs) or additionally presenting these symptoms after taking acetylsalicylic acid at the hospital. *p*‐value <0.05 was considered as statistically significant. The level of statistical significance after the Bonferroni correction of multiple testing is 0.05/21 = 0.0024 and is shown in **bold**.

In the N‐ERD group the proportion of patients born in winter, spring, summer and autumn was 1.5%, 1.4%, 1.3%, 1.5%, and in the CRS group 9.1%, 9.5%, 9.0%, 8.7%, respectively (Table S3). SOB was not associated with N‐ERD, CRS, CRSwNP, CRSsNP after Bonferroni correction of multiple testing (Table 1).

When stratified the models by the presence of either AR or asthma, being born in winter was associated with CRSwNP with AR (p = 0.014), as compared with being born in summer, yet this was not significant after Bonferroni correction (Table 2). No significant associations were observed in the CRS subgroups without asthma or AR (Table 2). Being born in autumn or winter was associated with combined NAR and AR (Table 2). Being born in winter or spring was associated with combined NAR and asthma (Table 2), whereas being born in autumn protected from NAR without asthma (Table 2). After Bonferroni correction the results were not significant.

**TABLE 2:** The association of season of birth with CRS, RARS and NAR.

Disease	Autumn		Winter		Spring	
	OR (95% CI)	*p*	OR (95% CI)	*p*	OR (95% CI)	*p*
Any CRS without asthma	0.97 (0.87–1.07)	0.523	0.98 (0.89–1.09)	0.717	1.04 (0.94–1.15)	0.430
Any CRS with asthma	0.98 (0.89–1.08)	0.690	1.04 (0.94–1.14)	0.445	1.07 (0.97–1.17)	0.161
CRSsNP without asthma	0.95 (0.84–1.08)	0.461	0.99 (0.88–1.12)	0.892	1.01 (0.89–1.13)	0.923
CRSsNP with asthma	0.93 (0.81–1.07)	0.288	1.02 (0.89–1.17)	0.773	1.06 (0.93–1.21)	0.354
CRSwNP without asthma	1.01 (0.85–1.2)	0.888	0.96 (0.81–1.15)	0.662	1.07 (0.91–1.26)	0.430
CRSwNP with asthma	1.06 (0.92–1.22)	0.445	1.08 (0.93–1.24)	0.310	1.03 (0.89–1.18)	0.719
N‐ERD without asthma	1.17 (0.72–1.88)	0.530	1.35 (0.85–2.14)	0.209	1.0 (0.61–1.63)	0.996
N‐ERD with asthma	1.15 (0.95–1.38)	0.155	1.17 (0.97–1.41)	0.105	1.08 (0.9–1.3)	0.427
Any CRS without AR	0.96 (0.89–1.03)	0.254	0.98 (0.91–1.06)	0.639	1.04 (0.97–1.12)	0.307
Any CRS with AR	1.09 (0.9–1.31)	0.404	1.2 (1.0–1.45)	0.053	1.17 (0.98–1.41)	0.089
CRSsNP without AR	0.94 (0.85–1.04)	0.216	0.99 (0.9–1.09)	0.831	1.01 (0.92–1.11)	0.868
CRSsNP with AR	0.96 (0.75–1.23)	0.744	1.1 (0.86–1.4)	0.444	1.19 (0.94–1.5)	0.144
CRSwNP without AR	1.0 (0.89–1.13)	0.983	0.98 (0.87–1.1)	0.685	1.03 (0.92–1.16)	0.586
CRSwNP with AR	1.35 (0.98–1.84)	0.063	1.47 (1.08–2.0)	0.014	1.14 (0.83–1.57)	0.414
N‐ERD without AR	1.1 (0.91–1.33)	0.320	1.13 (0.94–1.37)	0.195	1.07 (0.89–1.3)	0.460
N‐ERD with AR	1.42 (0.92–2.21)	0.115	1.53 (0.99–2.35)	0.055	1.04 (0.66–1.65)	0.864
Any asthma	1.06 (1.02–1.11)	0.003	1.08 (1.04–1.13)	**<0.0001**	1.04 (1.0–1.08)	0.051
Asthma with AR	1.16 (1.08–1.25)	**<0.0001**	1.2 (1.12–1.29)	**<0.0001**	1.13 (1.05–1.22)	0.001
Asthma without AR	1.01 (0.97–1.06)	0.519	1.02 (0.98–1.07)	0.360	1.0 (0.96–1.05)	0.871
AR	1.13 (1.06–1.21)	**<0.0001**	1.2 (1.12–1.28)	**<0.0001**	1.15 (1.08–1.22)	**<0.0001**
NAR	0.97 (0.9–1.05)	0.513	1.06 (0.98–1.14)	0.164	1.04 (0.97–1.13)	0.266
Combined NAR and AR	1.21 (1.02–1.43)	0.028	1.32 (1.12–1.55)	0.001	1.13 (0.95–1.33)	0.161
NAR without AR	0.92 (0.84–1.0)	0.058	0.99 (0.91–1.08)	0.847	1.02 (0.94–1.11)	0.592
NAR with asthma	1.06 (0.96–1.18)	0.261	1.12 (1.0–1.24)	0.043	1.12 (1.01–1.24)	0.036
NAR without asthma	0.88 (0.79–0.99)	0.034	0.99 (0.89–1.11)	0.883	0.97 (0.87–1.08)	0.531

*Note*: *p* values by binary logistic regression adjusted by sex and age. Winter was the Reference. CI = confidence intervals. Reference value (Ref) was summer (Jun‐Aug) in all models. The information extraction method from clinical texts was based on two separate methods that have been previously described.^6^ The level of statistical significance after the Bonferroni correction of multiple testing is 0.05/75 = 0.0007 and is shown in **bold.**

Overall this hospital cohort showed that being born in autumn or winter is associated with asthma and AR and being born in spring is additionally associated with AR when having summer as a reference. This is in part in line with previous studies in Finland showing that exposure to greenness during pregnancy increases the risk of developing asthma^7^

Interestingly our findings in a population‐based study have shown that adult‐onset asthma was positively associated with being born between January and June.^3^ This contrasting finding might be related to differences of study population and their birth decade, which might affect the risk. We have previously shown in a population‐based study that birth decade affects sensitization pattern and the asthma risk.^8^

Also in other countries the literature on the role of SOB in asthma and allergy is conflicting, which might in part be related to variation of the populations, allergens, pollutants, luminosity and other factors. In the European Community Respiratory Health Study information was collected randomly from 54 centers of 186,723 individuals.^9^ The study did not find significant effects in AR based on birth month, borderline reduced risks were detected in being born in September or October compared to January, which is in part in line to our observation, in which being born in summer (June‐August) protected from AR. The slightly different results might be explained by different population and luminosity of different parts of Europe as compared with Finland.

A Danish study compared data of asthma and/or AR subjects (*n* = 7796) obtained from general practices between June 1977 and June 1978, with controls of the entire Danish population (*N* = 5,095,969) and detected that AR patients born between February and May were overrepresented and those born between July and January were underrepresented compared to the controls, whereas the birth month distribution of asthma patients was similar to the controls.^10^ A study of US has evaluated the connection between several different diseases and the month of birth between years 1985–2013 (*N* = 1,749,400) and reported an association between asthma and the month of birth with peak occurrences of asthma cases in individuals born in June and October and smaller peaks in those born in August and September.^9^ A German birth cohort study (*n* = 1314, born in 1990), which is overrepresented by parental allergies, had 941 participants fulfilling 20‐year follow‐up.^11^ The study group showed that being born between June and November appeared to be associated with a slightly increased hazard of AR and AR with asthma compared to those born between December and May. The difference might be related to different population (young German population) and differences in the luminosity in Germany as compared with Finland.

Overall, our study suggests that being born in autumn or winter is associated with asthma and AR in Finland. This could reflect seasonal contrast in luminosity, vitamin D metabolism, greenness and of air pollutants affecting allergenicity, immunological responses the risk of AR.^12^ Our findings could also suggest that pathogenesis of CRS and N‐ERD are less related to these early life events, yet studies with increased population size are needed.

A limitation is that the study sample of the hospital cohort may not fully represent the entire population. There might be small gaps in the EHR entries of diagnoses that may lead to a presumed underrepresentation of diseases. On the other hand, this is likely to have affected the cohort regardless of the patient's SOB, and the diseases are physician‐diagnosed, mitigating this as a potential source of bias. We acknowledge that data was not available related to the following potential confounders: onset of the disease, socioeconomic status, race and different regions, which might mitigate the findings.

Still, our results strengthen the previous findings that early life events have a role in allergy related chronic airway diseases, and even with adult onset of them^13^ and highlights the need to focus also on early life factors in prevention of chronic inflammatory airway disease burden. As no association was observed between SOB and CRSsNP, CRSwNP, N‐ERD or NAR, further studies on this are warranted.

## AUTHOR CONTRIBUTIONS

All authors participated in the planning and conception of the study and the analytical strategy. RH, MN, AM and STS performed the data analyses and wrote the manuscript. All authors have assisted in data management, analyses and critical review of the manuscript.

## CONFLICT OF INTEREST STATEMENT

STS reports consultancies for AstraZeneca, Clario, Novartis, Sanofi Pharma, Roche Products, and a grant from GlaxoSmithKline. All are outside the submitted work. All other authors declare no conflicts of interest.

## CONSENT FOR PUBLICATION

Not applicable.

## Supporting information

Supporting Information S1

## Data Availability

Due to Finnish data protection legislation, confidential, health‐related data, the datasets produced and/or examined during this study are not accessible to the general public. They can solely be managed by designated individuals within the study group for specific research objectives. The datasets analyzed during the current study are available from the corresponding author upon reasonable request. Data use permissions can be applied from the competent authorities.
