# Supplementary material for: Being born in autumn or winter is associated with asthma and allergic rhinitis in Finland
Source: Clin Transl Allergy. 2024 Jul 19;14(7):e12383. doi: 10.1002/clt2.12383 (PMC11259556; doi:10.1002/clt2.12383)
Supplement: Supplementary file 1 — Supporting Information S1 [file CLT2-14-e12383-s001.docx]

**SUPPLEMENTARY MATERIAL:**

**Being born in autumn or winter is associated with asthma and allergic rhinitis in Finland**

Riikka Hänninen^1^, Aada Murtomäki^1,2^, Fanni Svärd^1,3^, Aarno Dietz^1,3^, Paulus Torkki^4^, Jari Haukka^4^, Mikko Nuutinen^1,2,5^* & Sanna Toppila-Salmi*^1,2,3,5^

^1^Department of Otorhinolaryngology, University of Eastern Finland Joensuu, Kuopio

^2^Inflammation Center, Skin and Allergy Hospital, Helsinki University Hospital and University of Helsinki, Hospital District of Helsinki and Uusimaa, Helsinki, Finland.

^3^Department of Otorhinolaryngology, Kuopio University Hospital, Wellbeing services county of North Savo, Kuopio, Finland

^4^Department of Public Health, University of Helsinki, Helsinki, Finland

^5^Haartman Institute, Medicum, PO Box 21 (Haartmaninkatu 3), 00014 University of Helsinki, Helsinki, Finland

*Shared last author

**Address correspondence and reprint requests to:**

Sanna Toppila-Salmi

MD PhD, Professor

1.)Department of Otorhinolaryngology, Kuopio University Hospital and University of Eastern Finland, PO Box 100, 70029, Kuopio, Finland

2.)Department of Allergy, Helsinki University Hospital and University of Helsinki, PO Box 160 (Meilahdentie 2), 4th fl. room 480, FI-00029 HUS, Finland

+358505431421,+358447172508, +358401581137

[sanna.salmi@uef.fi](mailto:sanna.salmi@uef.fi)

[sanna.salmi@helsinki.fi](mailto:sanna.salmi@helsinki.fi)

ORCID: 0000-0003-0890-6686

**Table S1**. The ICD-10 diagnoses that were used in data search in 2007-2019.

| A49.3 | Mycoplasma infection |
| --- | --- |
| B27 | Infectious mononucleosis. |
| B37 | Candidiasis |
| B44 | Aspergillosis |
| B95 | Streptococcus and staphylococcus as the cause of diseases classified to other chapters |
| D80-D84 | Immunodeficiencies |
| H05.01 | Cellulitis of orbit |
| H60 | Otitis externa |
| H65 | Nonsuppurative otitis media |
| H66 | Suppurative and unspecified otitis media |
| J01 | Acute sinusitis |
| J02 | Acute pharyngitis |
| J03 | Acute tonsillitis |
| J04 | Acute laryngitis and tracheitis |
| J18 | Pneumonia, unspecified organism. |
| J21 | Acute bronchiolitis |
| J30 | Vasomotor and allergic rhinitis |
| J31 | Chronic rhinitis |
| J32 | Chronic sinusitis |
| J33 | Nasal polyp |
| J34.2 | Deviated nasal septum |
| J35 | Chronic diseases of tonsils and adenoids |
| J36 | Peritonsillar abscess |
| J37 | Chronic laryngitis and laryngotracheitis |
| J38 | Diseases of vocal cords and larynx, not elsewhere classified |
| J38.3 | VCD |
| J40 | Bronchitis, not specified as acute or chronic |
| J45 | Asthma |
| J46 | Status asthmaticus |
| J47 | Bronchiectasis |
| J67 | Hypersensitivity pneumonitis |
| J82 | Pulmonary eosinophilia, not elsewhere classified |
| K02 | Caries |
| K04 | Diseases of pulp and periapical tissues |
| K05 | Gingivitis and periodontal diseases |
| K11.2 | Sialoadenitis |
| K11.5 | Sialolithiasis |
| L02.11 | Cutaneous abscess of neck |
| L20 | Atopic dermatitis |
| L50 | Urticaria |
| Q17.5 | Otapostasis |
| Q18.0 | Sinus, fistula and cyst of branchial cleft |
| Q38.1 | Ankyloglossia |
| R04.0 | Epistaxis |
| R06.5 | Mouth breathing |
| T17 | Foreign body in respiratory tract |
| T18.1 | Foreign body in esophagus |
| T78 | Anaphylaxis, Angioedema, Allergy NAS |
| Z51.6 | desensitization to allergens |
| Z88. 6 | Allergy status to analgesic agent |
| Z91.01 | Food allergy |

**Table S2.** Key words of N-ERD for the mining of patient variables from the clinical text. Specific dictionaries for evaluating when matched key words relate to the negation of disease, family history or good medical status (4).

| **Disease** | **Key word** | **Rule based dictionary** |
| --- | --- | --- |
| N-ERD | aerd | no |
|  | samter | no |
|  | aspirin | no |
|  | asa | no, mg |

**Table S3.** The proportion of subjects being born in each season and having different diseases

| **Season of birth** | **Winter** | **Spring** | **Summer** | **Autumn** |
| --- | --- | --- | --- | --- |
| Number of patients | 18150 | 19688 | 18980 | 18050 |
| AR | 2279 (12.56) | 2356 (11.97) | 2028 (10.68) | 2185 (12.11) |
| NAR | 1380 (7.6) | 1483 (7.53) | 1374 (7.24) | 1266 (7.01) |
| Asthma | 7817 (43.07) | 8294 (42.13) | 7809 (41.14) | 7704 (42.68) |
| any_CRS | 1649 (9.09) | 1865 (9.47) | 1712 (9.02) | 1570 (8.7) |
| CRSsNP | 948 (5.22) | 1060 (5.38) | 989 (5.21) | 879 (4.87) |
| CRSwNP | 655 (3.61) | 718 (3.65) | 668 (3.52) | 652 (3.61) |
| N-ERD | 278 (1.53) | 271 (1.38) | 246 (1.3) | 263 (1.46) |
| Any CRS without Asthma | 773 (23.88) | 886 (27.37) | 825 (25.49) | 753 (23.26) |
| Any CRS with Asthma | 876 (24.61) | 979 (27.51) | 887 (24.92) | 817 (22.96) |
| CRSsNP without Asthma | 513 (24.32) | 566 (26.84) | 542 (25.7) | 488 (23.14) |
| CRSsNP with Asthma | 435 (24.62) | 494 (27.96) | 447 (25.3) | 391 (22.13) |
| CRSwNP without Asthma | 248 (23.11) | 297 (27.68) | 270 (25.16) | 258 (24.04) |
| CRSwNP with Asthma | 407 (25.12) | 421 (25.99) | 398 (24.57) | 394 (24.32) |
| N-ERD without Asthma | 41 (29.08) | 33 (23.4) | 32 (22.7) | 35 (24.82) |
| N-ERD with Asthma | 237 (25.85) | 238 (25.95) | 214 (23.34) | 228 (24.86) |
| Any CRS without AR | 1410 (23.98) | 1611 (27.39) | 1504 (25.57) | 1356 (23.06) |
| Any CRS with AR | 239 (26.12) | 254 (27.76) | 208 (22.73) | 214 (23.39) |
| CRSsNP without AR | 813 (24.36) | 901 (27.0) | 861 (25.8) | 762 (22.83) |
| CRSsNP with AR | 135 (25.05) | 159 (29.5) | 128 (23.75) | 117 (21.71) |
| CRSwNP without AR | 557 (23.67) | 635 (26.99) | 598 (25.41) | 563 (23.93) |
| CRSwNP with AR | 98 (28.82) | 83 (24.41) | 70 (20.59) | 89 (26.18) |
| N-ERD without AR | 227 (25.59) | 233 (26.27) | 211 (23.79) | 216 (24.35) |
| N-ERD with AR | 51 (29.82) | 38 (22.22) | 35 (20.47) | 47 (27.49) |
| Any asthma | 7817 (24.72) | 8294 (26.23) | 7809 (24.69) | 7704 (24.36) |
| Astma with AR | 1809 (25.75) | 1841 (26.21) | 1601 (22.79) | 1773 (25.24) |
| Asthma without AR | 6008 (24.42) | 6453 (26.23) | 6208 (25.24) | 5931 (24.11) |
